# Dapagliflozin has No Protective Effect on Experimental Pulmonary Arterial Hypertension and Pulmonary Trunk Banding Rat Models

**DOI:** 10.3389/fphar.2021.756226

**Published:** 2021-11-01

**Authors:** Huayang Li, Yitao Zhang, Shunjun Wang, Yuan Yue, Quan Liu, Suiqing Huang, Huajing Peng, Yi Zhang, Weijie Zeng, Zhongkai Wu

**Affiliations:** ^1^ Department of Cardiac Surgery, The First Affiliated Hospital of Sun Yat-Sen University, Guangzhou, China; ^2^ NHC Key Laboratory of Assisted Circulation (Sun Yat-sen University), Guangzhou, China; ^3^ Department of Cardiovascular, The Sixth Affiliated Hospital of Sun Yat-sen University, Guangzhou, China

**Keywords:** pulmonary trunk banding, pulmonary vascular remodeling, right ventricular dysfunction, SGLT2 inhibitors, dapagliflozin, pulmonary arterial hypertension

## Abstract

Sodium-glucose cotransporter-2 (SGLT2) inhibitors, a novel class of hypoglycemic drugs, show excellent cardiovascular benefits, and have further improved heart failure outcomes, significantly reducing cardiovascular and all-cause mortality irrespective of diabetes status. However, the efficacy of SGLT2 inhibitors in pulmonary arterial hypertension (PAH) and right ventricular (RV) dysfunction remains unknown. This study aimed to evaluate the effects of dapagliflozin in rats with PAH and RV dysfunction. PAH was induced in rats by monocrotaline (MCT) subcutaneous injection (60 mg/kg). Isolated RV dysfunction was induced in another group of rats by pulmonary trunk banding (PTB). Dapagliflozin (1.5 mg/kg) was administered daily *via* oral gavage one day (prevention groups) or two weeks (reversal groups) after modeling. Echocardiography and hemodynamic assessments were used to observe pulmonary vascular resistance and RV function. Histological staining was used to observe pulmonary vascular and RV remodeling. As compared with MCT group, dapagliflozin treatment did not significantly improve the survival of rats. Pulmonary arterial media wall thickness in MCT group was significantly increased, but dapagliflozin did not significantly improved vascular remodeling both in the prevention group and reversal group. In MCT group, RV hypertrophy index, RV area, the fibrosis of RV increased significantly, and RV function decreased significantly. Consistently, dapagliflozin did not show protective effect on the RV remodeling and function. In the PTB model, we also did not find the direct effect of dapagliflozin on the RV. This is a negative therapeutic experiment, suggesting human trials with dapagliflozin for PAH or RV failure should be cautious.

## Introduction

Pulmonary arterial hypertension (PAH) is defined as a resting mean pulmonary artery pressure of 25 mmHg or above, and its prevalence varies from 11 to 26 cases per million adults globally (Thenappan et al., 2018). The pathological changes of PAH are characterized by proliferative and obstructive remodeling of the pulmonary arteries, together with excessive vasoconstriction and increased pulmonary vascular resistance (PVR), leading to right ventricular (RV) hypertrophy and dysfunction (Humbert et al., 2019). Fourteen specific medications are currently available for PAH (Tuder et al., 2013), however, they all focus on dilating the partially occluded vessels and none of them target the adverse vascular remodeling. More unfortunately, possible direct effects of potential therapies on the RV are also rarely assessed, despite the recognized importance of RV function in the overall prognosis of PAH (Voelkel et al., 2012). Thus, PAH patients continue to face frequent hospitalizations, poor health status, high medical costs, and high residual risks of premature mortality. Despite adequate treatments, the 3-years survival rate of PAH patients is estimated to be between 54 and 84.4% (Jang and Chung, 2019). Collectively, there is an imperious demand for novel medications which can efficiently target the pathological mechanisms that cause the progression of PAH.

Sodium-glucose cotransporter-2 (SGLT2) inhibitors, which act independently of insulin, represent newly developed oral antidiabetic drugs that are practiced for type 2 diabetes mellitus (T2DM) management (Yaribeygi et al., 2020). The currently marketed SGLT2 inhibitors include dapagliflozin, empagliflozin, canagliflozin, ertugliflozin, ipragliflozin, tofogliflozin, etc. Apart from lowering blood glucose, dapagliflozin has recently been shown to have positive effects on patients with heart failure (McMurray et al., 2019). Correspondingly, the United States Food and Drug Administration (FDA) approved dapagliflozin as the first SGLT2 inhibitors to reduce the risk of cardiovascular death and hospitalization for heart failure in adult patients with or without T2DM in 2020 (U. S. Food and Drug Administration, 2020). Gratifyingly, empagliflozin was recently found to reduce the mortality and prevent the progression of pulmonary vasculopathies in PAH rats induced by monocrotaline (MCT), although the underlying mechanisms had not been clarified (Chowdhury et al., 2020). But the direct effects of empagliflozin on the RV remodeling and dysfunction were not further studied in the above article. Besides experimental PAH, data from human studies demonstrated that empagliflozin produced rapid reduction in pulmonary artery (PA) pressure (began at week 1 and amplified over time) in patients with heart failure (Nassif et al., 2021). However, different SGLT2 inhibitors have different off-target effects (Mancini et al., 2018; Behnammanesh et al., 2020), meaning that even though empagliflozin has protective effects on PAH, other SGLT2 inhibitors may not.

While dapagliflozin has been reported to significantly lower RV systolic pressure (RVSP) during exercise in patients with T2DM and cardiovascular risk (Kayano et al., 2020), the effects of dapagliflozin on pulmonary vascular remodeling and RV remodeling are largely unknown. We therefore set out to explore the effects of dapagliflozin on PAH induced by MCT and isolated RV pressure overload induced by pulmonary trunk banding (PTB). Although many positive effects have been described in previous prevention studies, to date few effective medicines for animals with established advanced PAH have been described, so we also started to administer dapagliflozin in the middle of the disease stimulation.

Given the profound cardiovascular benefits observed in SGLT2 inhibitors, we speculated that dapagliflozin would prevent and reverse MCT-induced pathological pulmonary vascular changes, and alleviate pressure overload-induced RV remodeling and dysfunction.

## Materials and Methods

### Animals and Drug Administration

**FIGURE 1:**
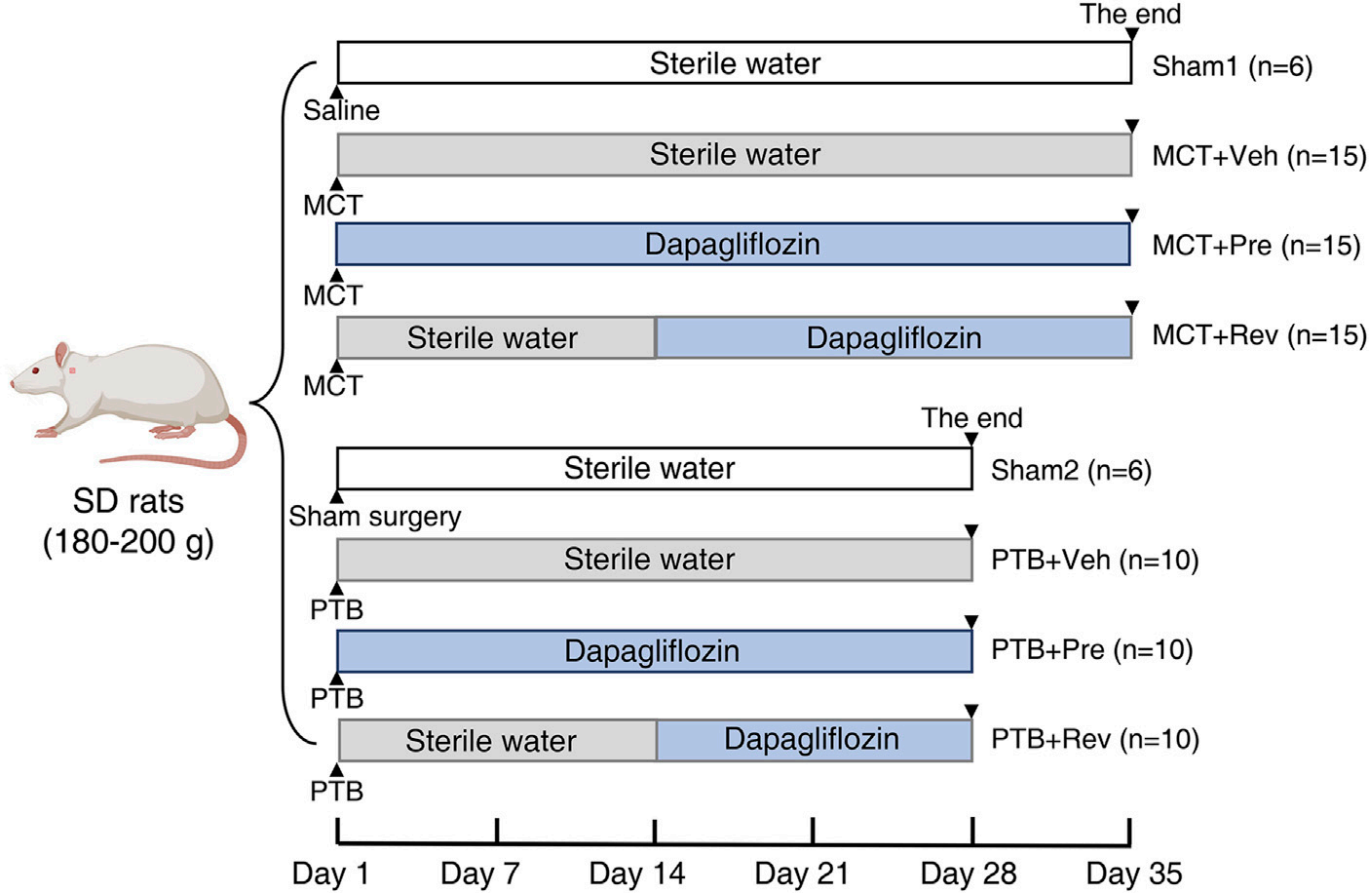
Study design. In MCT study, rats were subcutaneous injected MCT (60 mg/kg) or equal volume of saline on Day 0. In PTB study, rats were performed PTB surgery or sham surgery on Day 0. Rats were administered dapagliflozin (1.5 mg/kg/day) or equal volume of sterile water by oral gavage.

Males Sprague-Dawley rats (180–200 g, purchased from the Laboratory Animal Center of Sun Yat-sen University, Guangzhou, China) were housed in a specific pathogen free room with a 12 h light-dark cycle at 22 ± 2°C. Water and rat chow were provided ad libitum. The effects of dapagliflozin on the development of PAH and the following RV remodeling were evaluated in the MCT model. Rats were randomized to sham1 (*n* = 6), MCT + vehicle (*n* = 15), MCT + prevention (*n* = 15), and MCT + reversal groups (*n* = 15). In order to separate the pulmonary vascular effects of dapagliflozin from the direct RV effects, PTB model was further applied. For this part of the study, rats were randomized to sham2 (*n* = 6), PTB + vehicle (*n* = 10), PTB + prevention (*n* = 10), and PTB + reversal groups (*n* = 10). For the two prevention groups, rats were administered dapagliflozin (1.5 mg/kg/day) by oral gavage one day after modeling for 5 weeks in PAH study and 4 weeks in PTB study. For the two reversal groups, rats were administered equal amount of sterile water by oral gavage one day after modeling for two weeks and then dapagliflozin (1.5 mg/kg/day) by oral gavage for 3 weeks in PAH study and 2 weeks in PTB study. Rats in the sham and vehicle groups were administered equal amount of sterile water by oral gavage for 5 weeks in PAH study and four 4 in PTB study (Figure 1).

### Pulmonary Arterial Hypertension

Pulmonary arterial hypertension in rats was induced by MCT subcutaneous injection. Briefly, MCT (Topscience, Shanghai, China) was weighed, dissolved in hydrochloric acid, titrated with sodium hydroxide solution until the pH value of solution was equal to 7.35–7.45, and finally set the MCT concentration to 20 mg/ml with saline. Then sterile syringe filters (SLGP033RB, Merck Millipore, Darmstadt, Germany) was used to filter and sterilize the MCT solution. After the rats were weighed, MCT solution (60 mg/kg) was injected under the skin of the back of the rats’ neck.

### Pulmonary Trunk Banding

Briefly, the rats were anesthetized with pentobarbital (50 mg/kg) *via* intraperitoneal injection. After endotracheal intubation with a 16-gauge tube, the rats were mechanically ventilated using a ventilator (RoVent Jr, Kent Scientific Corporation, Torrington, CT). Disinfected with iodophor three times, the skin was incised and the second intercostal space was opened. Then the pulmonary artery was dissected free from the adipose tissue and aorta. The pulmonary trunk was banded with 4–0 silk suture and the suture was tied tightly against an 18-gauge needle which was removed quickly to produce a fixed constriction of 1.2 mm in diameter. Rats in the sham group underwent the same procedures except for tying the pulmonary trunk. After the operation was completed, the rats were placed on a heating blanket to maintain body temperature until awake.

### Echocardiography

Vevo 2100 (VisualSonics, Toronto, Canada) with a 25 MHz linear array transducer was used for transthoracic echocardiography. During operation, the rats were anesthetized with pentobarbital sodium (50 mg/kg, i.p.). Short axis M-mode recordings were obtained to measure left ventricle ejection fraction (LVEF). The pulsed-wave doppler recording at the right ventricular overflow tract was used to measure pulmonary acceleration time (PAT). RV area was obtained from two-dimensional apical four-chamber view. RV function was assessed by tricuspid annular plane systolic excursion (TAPSE).

### RV Catheterization and Hypertrophy Index

After echocardiography, terminal invasive hemodynamic measurements were performed to measure RV pressure *via* RV catheterization. The rats were anaesthetized with pentobarbital sodium (50 mg/kg, i.p.) and fixed on a plank. The skin and tissues of the right neck were incised with lidocaine for local analgesia, and then the right jugular vein was isolated. After heparinization *via* the right jugular vein, the PE-50 tube filled with heparin saline was connected to a pressure sensor (Techman, Chengdu, China) and inserted into the right external jugular vein. The appearance of the ventricular pressure wave indicated that the catheter reached the RV. Then the RV pressure was recorded and the RVSP was analyzed. After the RVSP measurements, the chest was quickly opened to harvest the hearts and lungs under anesthetization. The left and right atrium and blood vessels along the junction of the atrioventricular compartment were cut. Then the RV, interventricular septum (IVS), and left ventricle (LV) were separated and weighted separately. The RV hypertrophy index was calculated as [RV/(IVS + LV)].

### Masson Staining and Hematoxylin-Eosin (H and E) Staining

After hemodynamic measurements and blood withdrawals, the hearts and lungs were excised and harvested for fibrosis, morphometric and histologic analysis. The isolated heart and middle lobe of the right lung were fixed in 4% paraformaldehyde for 24 h, then they were embedded in paraffin and sectioned into 5-μm-thick slices. The heart slices were stained with Masson’s trichrome to determine the RV collagen volume fraction, and the lung slices were stained with H and E dyes to determine the arteriole remodeling. Digital light microscope (Olympus, Tokyo, Japan) was used for overall histological assessment. For quantitative analysis, pictures were selected from each section by an investigator who was unaware of the grouping. The fibrosis of RV was assessed by Image-Pro Plus software (Version 6.0, Media Cybernetics, Silver Springs, MD, United States). Pulmonary arterial medial wall thickness (WT) was calculated by the following formula: WT (%) = area_ext_-area_int_/area_ext_ × 100, where area_ext_ and area_int_ were areas bounded by external and internal elastic lamina, respectively.

### Statistical Analysis

All values were presented as mean ± SEM. Analyses were done with GraphPad Prism 9 (GraphPad Software Inc., La Jolla, CA, United States). Student unpaired *t* test was performed to compare means between 2 groups. Comparisons between multiple groups were made with One-way analysis of variance (ANOVA) followed by Tukey’s text for post hoc comparisons. Statistical significance was defined as *p* < 0.05.

## Results

### Dapagliflozin Cannot Reduce the Mortality of MCT Rats

**FIGURE 2:**
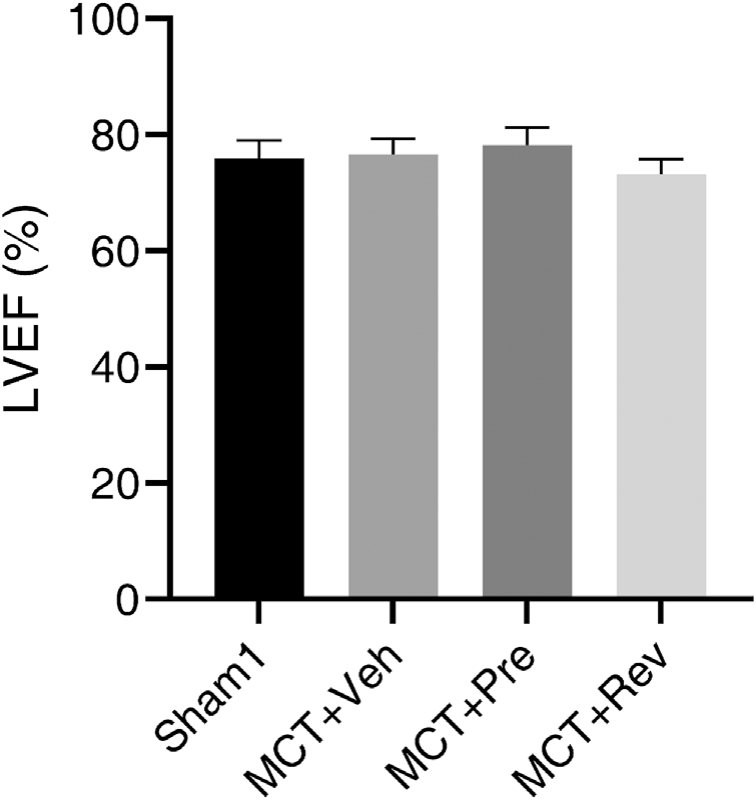
Neither MCT or dapagliflozin had any effect on the LV systolic function of the rats. Short axis M-mode recordings were obtained to measure LVEF five weeks after MCT injection. (*n* = 6 for Sham1, *n* = 10 for MCT + vehicle, MCT + prevention, and MCT + reversal).

**FIGURE 3:**
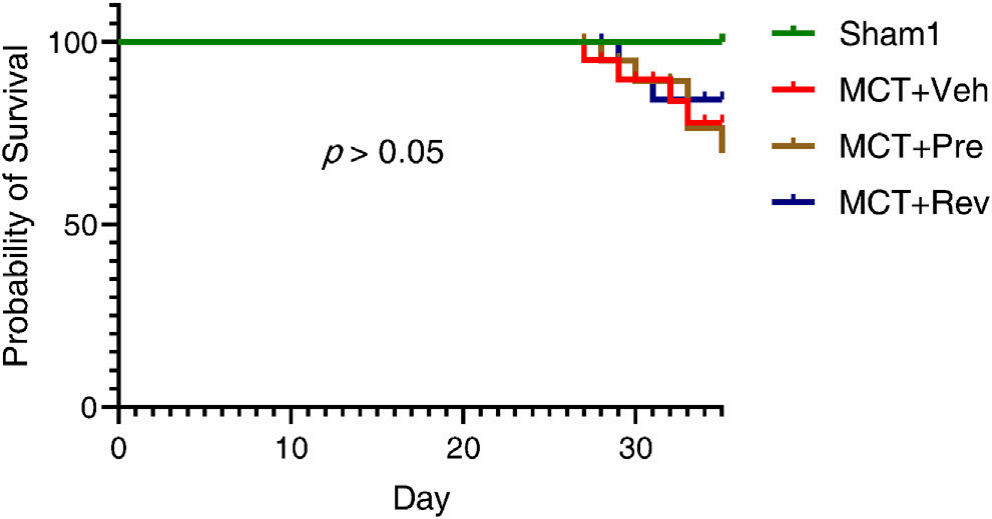
Dapagliflozin could not reduce the mortality of MCT rats. Observed for five weeks after MCT subcutaneous injection. (*n* = 6 for Sham1, *n* = 15 for MCT + vehicle, MCT + prevention, and MCT + reversal).

At the end of the experiment, neither MCT injection or dapagliflozin administration had any effect on the LV systolic function of the rats (Figure 2). During the five-weeks observation period, four rats died in MCT + vehicle group, five rats died in MCT + prevention group, and three rats died in MCT + reversal groups. But the autopsy of dead rats did not find a clear cause of death. There was no statistical difference in the survival curve between the three groups of MCT (Figure 3).

### Dapagliflozin has No Effect on Pulmonary Vascular Remodeling in MCT Rats

**FIGURE 4:**
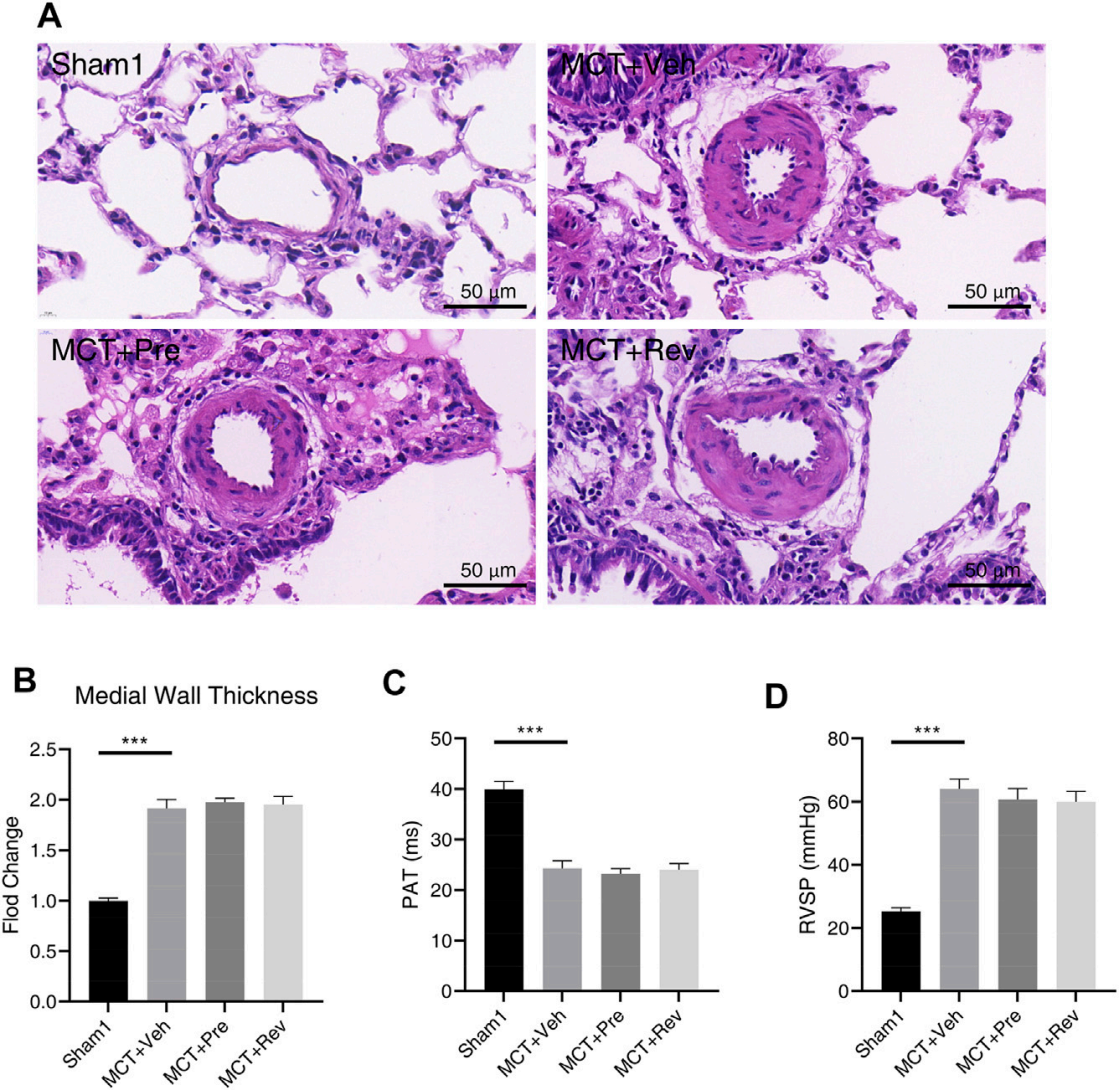
Dapagliflozin could not alleviate pulmonary vascular remodeling in MCT rats. **(A)** Representative images of H and E staining in the lung sections; Quantification of medial wall thickness **(B)**, PAT**(C)**, RVSP **(D)** five weeks after MCT injection. (***p < 0.001; *n* = 6 for Sham1, *n* = 10 for MCT + vehicle, MCT + prevention, and MCT + reversal).

After subcutaneous injection, the MCT alkaloid is activated to the dehydromonocrotaline (MCTP) in the liver by cytochrome P-450 (Reid et al., 1998). Then MCTP causes endothelial cell damage and pulmonary artery smooth muscle cells (PASMCs) proliferation, which leads to pulmonary arterial medial hypertrophy and obstructive pulmonary vascular remodeling (Gomez-Arroyo et al., 2012). Lung sections were stained with H and E to evaluate the severity of the pulmonary vascular disease. As illustrated in Figures 4A,B, WT increased significantly five weeks after MCT injection. However, dapagliflozin couldn’t attenuate pulmonary vascular remodeling. The obstruction of the pulmonary vascular led to an increase in PVR, manifested by decreased PAT (Figure 4C) and increased RVSP (Figure 4D). Correspondingly, dapagliflozin administration also didn’t prolong PAT or decrease RVSP.

### Dapagliflozin Cannot Attenuate RV Remodeling or Improve RV Dysfunction in MCT Rats

**FIGURE 5:**
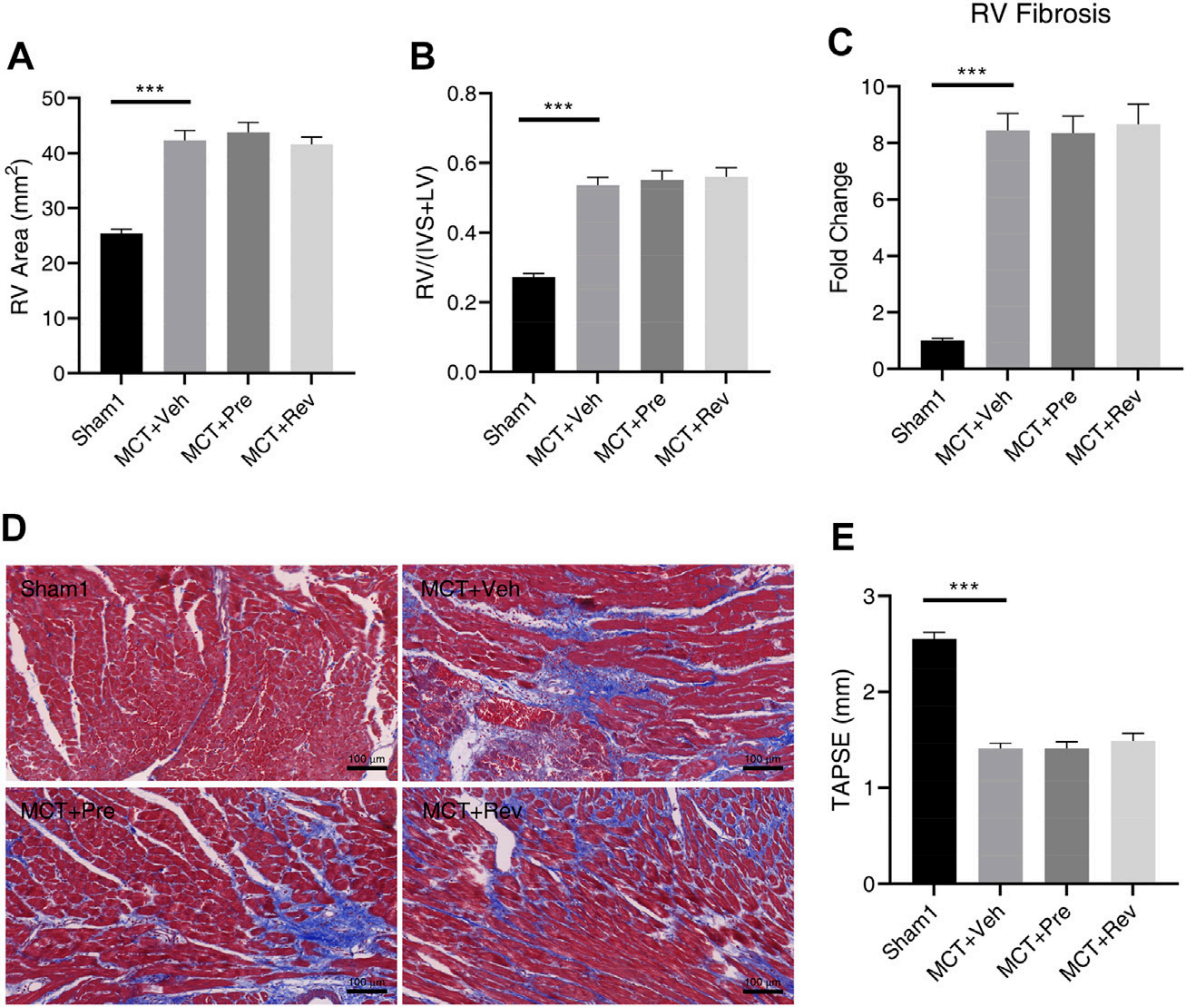
Dapagliflozin could not attenuate RV remodeling or improve RV dysfunction in MCT rats. Quantification of RV area **(A)**, RV/(IVS + LV) **(B)**, RV fibrosis **(C)**, and TAPSE **(E)** five weeks after MCT injection; **(D)** Representative images of Masson staining in the cardiac sections. (***p < 0.001; *n* = 6 for Sham1, *n* = 10 for MCT + vehicle, MCT + prevention, and MCT + reversal).

Pressure overload leads to heart concentric hypertrophy to maintain cardiac output in the early stage, however, when the overload persists, the compensatory process may deteriorate into eccentric hypertrophy and culminates in cardiac dysfunction in the later stage (Pitoulis and Terracciano, 2020). As shown in Figure 5A, rats in MCT groups had enlarged RV chamber. But there was no statistical difference in the RV area between the MCT + vehicle group, MCT + prevention group, and MCT + reversal group. Although the cardiomyocytes are terminally differentiated cells and lose their ability of proliferation, they could increase their volume and muscle mass by hypertrophic remodeling to enhance the contractility under pressure overload (Hill and Olson, 2008). Five weeks after MCT injection, increased RV hypertrophy index confirmed the above theory (Figure 5B). Still, dapagliflozin couldn’t delay or reverse the hypertrophy of cardiomyocytes. Under various pathological stimuli, the heart will adjust its components to maintain heart function, and the most important thing is the change of collagen fibers. Of note, excessive fibrosis will further worsen heart function, leading to decreased contractility, stiffness of the ventricular wall, arrhythmia, etc. (Li et al., 2018). Collagen fibers can be stained blue by Masson staining, and then we found an 8.45-fold increase in the volume fraction of fibrosis in the RV in MCT + vehicle group compared to Sham1 group. Yet dapagliflozin treatment didn’t reduce the deposition of collagen fibers (Figures 5C,D).

Increase in afterload, enlargement of the RV chamber, hypertrophy of myocardial cells, and fibrosis of the RV finally led to the RV dysfunction (Figure 5E). Since dapagliflozin could neither alleviate PVR nor RV remodeling, no improvement in RV dysfunction was observed in MCT + prevention and MCT + reversal groups relative to MCT + vehicle group.

### Dapagliflozin Cannot Attenuate RV Remodeling or Improve RV Dysfunction in PTB Rats

**FIGURE 6:**
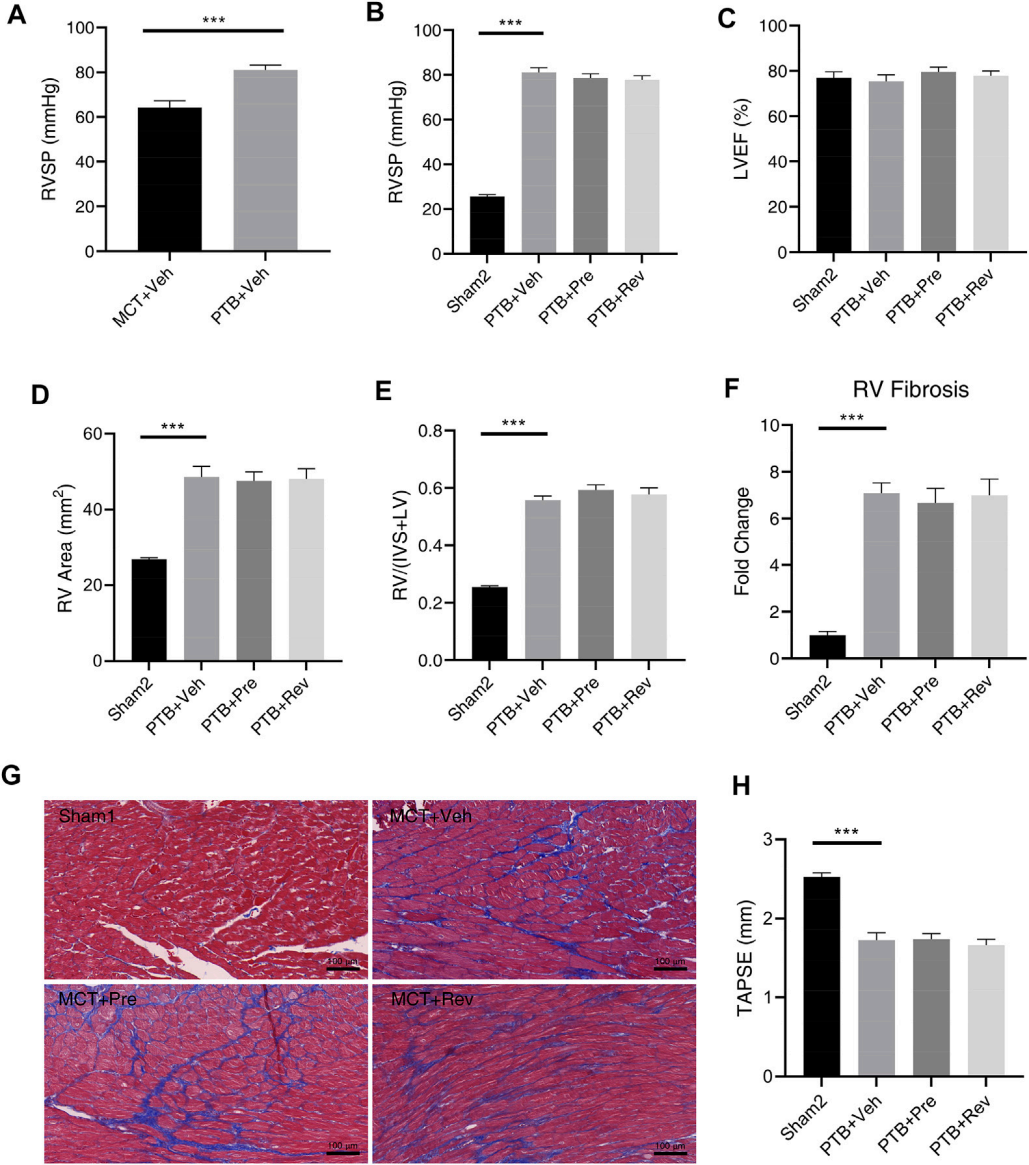
Dapagliflozin could not attenuate RV remodeling or improve RV dysfunction in PTB rats. **(A)** RVSP five weeks after MCT injection or four weeks after PTB surgery (n = 10 for two groups); Quantification of RVSP **(B)**, LVEF **(C)**, RV area **(D)**, RV/(IVS + LV) **(E)**, RV fibrosis **(F)**, and TAPSE **(H)**; **(G)** Representative images of Masson staining in the cardiac sections. (***p < 0.001; *n* = 6 for Sham2, *n* = 10 for PTB + vehicle, PTB + prevention, and PTB + reversal).

In the presence of pulmonary vascular disease, the RV with increased pressure overload is prone to develop RV insufficiency (Bogaard et al., 2009). Therefore, although dapagliflozin could not improve the RV remodeling and dysfunction in PAH rats, it may have a beneficial effect on RV in PTB rats. Unlike the MCT rats whose PVR increases slowly from the normal range, the PTB rats have a sudden increase in RV afterload after PTB surgery and then slowly increase as the rats grow up. RVSP was remarkably higher in PTB + vehicle rats as compared with MCT + vehicle rats (Figure 6A), but no rat died before the end of the experiment in PTB study.

Four weeks after PTB surgery, rats in different PTB groups all had similar RVSP (Figure 6B) and PTB surgery had no effect on LV systolic function (Figure 6C). Like dapagliflozin’s disappointing effects on MCT rats, dapagliflozin had no effect on RV in PBT rats. Poor RV remodeling and decreased RV systolic function had not been improved by dapagliflozin administration (Figures 6D–H).

## Discussion

Given the widespread clinical use of SGLT2 inhibitors and the likelihood that patients treated with these agents could develop conditions of further afterload stress, we aimed to better define the effects of dapagliflozin on pulmonary vascular remodeling, RV remodeling, and RV dysfunction *in vivo*. What we found, sadly, was that dapagliflozin could neither alleviate pulmonary vascular remodeling nor reduce PVR in PAH rats induced by MCT. More importantly, dapagliflozin did not appear to improve RV remodeling or dysfunction under afterload stress with or without pulmonary angiopathy.

Although PAH is divided into five clinical classifications and MCT-induced PAH in rats does not fully recapitulate the progression of PAH pathology in patients, classic MCT model could be used to efficiently reproduce the pathophysiology of PAH and to assess treatment efficacy in preclinical studies (Nogueira-Ferreira et al., 2015; Simonneau et al., 2019). Five weeks after MCT injection, obstructive pulmonary vascular remodeling and increased PVR were successfully verified which were not significantly reduced by dapagliflozin treatment in our study. Cumulative data from this model confirmed that uncontrolled proliferation and resistance to apoptosis of PASMCs are the main causes of pulmonary vascular obstruction (Gomez-Arroyo et al., 2012). Recently canagliflozin, but not dapagliflozin or empagliflozin, was found to inhibit the proliferation and migration of rat or human aortic vascular smooth muscle cells by stimulating the expression of heme oxygenase-1 (Behnammanesh et al., 2020). Empagliflozin was also found to increase apoptosis and reduce proliferation of PASMCs in rats’ pulmonary arterioles (Chowdhury et al., 2020), but we found that dapagliflozin couldn’t. This means that even though different SGLT2 inhibitors have similar structures, they have different pleiotropic effects. And the indications of one certain SGLT2 inhibitor cannot be simply extrapolated to other SGLT2 inhibitors.

Cardiac fibrosis is a common pathophysiologic companion of most myocardial diseases irrespective of its etiology. The accumulation of extracellular matrix and fibroblasts may have some protective effects in certain situations, but prolonged fibrosis usually leads to systolic and diastolic dysfunction, arrhythmogenesis, and adverse outcome (Fu et al., 2020). Current notion suggests that the most important determinant of longevity in PAH patients is not the severity of the vascular pathology, as previously assumed, but rather the RV function (D'Alonzo et al., 1991). And as neither a persistent reversal of pulmonary vascular remodeling nor a lasting reduction of the pulmonary resistance cannot be realized in PAH patients by currently available vasodilator drugs, an efficient specific cardioprotective medicine that can improve RV dysfunction despite elevated RV afterload may improve the quality of life and prolong lifespan of PAH patients. Sadly, dapagliflozin didn’t show any effects on RV remolding or dysfunction of MCT rats in our study.

RV pressure overload alone (PTB) is insufficient to induced RV failure, whereas in the context of PAH, RV failure happens with myocardial apoptosis and decrease of RV capillary density (Bogaard et al., 2009). What we found that no rats died during the observation period in PTB groups, although the PTB rats had a higher RVSP compared to the MCT rats, also validated the above view. So, the negative effect of dapagliflozin on RV remodeling and dysfunction in MCT rats may be explained by the fact that mediators released from altered pulmonary vascular in PAH interfere with adaptive RV response which already maximally challenged to meet the increased mechanical stress (Dupuis et al., 1996; Langleben et al., 2008). To investigate the role of dapagliflozin in isolated RV pressure overload which also occurs in patients with RV outflow tract obstruction, pulmonary valve stenosis, or pulmonary embolism, the PTB model was further applied. However, compared to the protective effects of dapagliflozin on the LV (McMurray et al., 2019; Shi et al., 2019), we obtained frustrating results. The efficacy of dapagliflozin on the LV possibly be based on its ability to decrease volume load of the LV by diuresis and natriuresis, and to decrease pressure load of the LV by reducing systemic arterial blood pressure independent of their glucose-lowering effect (Verma et al., 2017; Sternlicht and Bakris, 2019). Besides the two main and more certain indirect effects mentioned above, SGLT2 inhibitors also have direct effects on the LV, such as reducing inflammation, oxidative stress, and so on, although the underlying mechanisms have not been defined (Bonnet and Scheen, 2018; Yaribeygi et al., 2019). Undoubtedly, large similarities exist between the LV and RV. Of note, there are still important differences at the cellular and molecular levels in the LV versus RV responses to afterload increase as the different embryological origin of two ventricles and the different genes regulating energy production, reactive oxygen species production, antioxidant protection, and angiogenesis of them (Reddy and Bernstein, 2015). From an external perspective, the RV may be subjected to a ≥2-fold increase in afterload, whereas the afterload increase is usually <50% for the LV. In summary, the response of the LV to drugs cannot be extrapolated to the RV for granted. A growing body of clinical trials found that effective classic heart failure medicines (β-blockers, angiotensin-converting enzyme inhibitors, angiotensin II receptor blockers, etc.), developed and tested in patients with LV failure, could not improve RV dysfunction or survival in patients with RV failure (Winter et al., 2009).

The animal models we used are classic and commonly used (Nogueira-Ferreira et al., 2015; Cops et al., 2019), and the dose of dapagliflozin was also sufficient (Obermeier et al., 2010), but we had not seen any trend of disease remission. Therefore, the negative treatment effect of dapagliflozin we observed in this study cannot be interpreted as a statistical Type II error.

It takes a lot of time and manpower for a compound to go from animal experiments to clinical trials, and eventually become an effective drug for a certain disease, and this process is costly and has a high rate of failure. Indeed, while a drug is possible to “cure” many forms of PAH in animal models, the clinical profile of PAH patients is more resistant to therapy possibly because of the differences of the patients’ gender, age, diet, underlying diseases, histopathology and so on. The truth is that literatures are often biased toward positive results, while negative studies are underreported. Therefore, it is likely that other researchers might have the same findings as us, but because the results did not meet expectations, they chose not to publish the article. In other words, even though our results are depressing, it could make other researchers realize that dapagliflozin has no effect on PAH and RV dysfunction, so they may give up exploring the effects of dapagliflozin on PAH and RV dysfunction, and turn to find other more effective drugs.

### Limitations

Two potential limitations need to be addressed here. 1) We had not studied the effects of other SGLT2 inhibitors, such as empagliflozin, canagliflozin, ertugliflozin, etc., on experimental PAH or isolated RV dysfunction. Now we are conducting experiments on the treatment of PAH with empagliflozin and empagliflozin combined with other classic anti-PAH drugs, which may be able to make up for the deficiencies of this study. 2) We just used one dose of dapagliflozin (1.5 mg/kg/day) for treatment. However, in recent preclinical studies on the cardiovascular protective effects of dapagliflozin, researchers also only used dose of 1.5 mg/kg/day for treatment (Arow et al., 2020; Withaar et al., 2021). According to the manufacturer’s instructions and Obermeier’s study (Obermeier et al., 2010), dose of 1.5 mg/kg/day in male rats is equivalent to about 9–19 times the human clinical dose of 10 mg/day calculated according to area under the concentration/time curve. Therefore, the negative results of dapagliflozin on PAH and RV dysfunction should not be attributed to the underdosing of dapagliflozin in the present study.

## Conclusion

In the present work, we found that dapagliflozin could not reduce pulmonary vascular remodeling in rats with PAH, nor could it improve the remodeling and dysfunction of the RV under pressure overload with or without pulmonary angiopathy. Therefore, dapagliflozin may be used safely in this patient population when they are clinically indicated to reduce blood glucose level or risk of cardiovascular death, but should not be prescribed as a specific treatment for PAH or RV failure Jabbour et al., 2018; Kohler et al., 2017; Qiu et al., 2017; Scheen, 2019.

## Data Availability

The raw data supporting the conclusions of this article will be made available by the authors, without undue reservation.
